# Respuesta sobre la regulación colombiana de los productos bioterapéuticos

**DOI:** 10.26633/RPSP.2017.135

**Published:** 2017-09-11

**Authors:** Claudia Vaca González, Carolina Gómez, Alejandro Gaviría

**Affiliations:** 1 Departamento de Farmacia Universidad Nacional de Colombia Colombia Departamento de Farmacia, Universidad Nacional de Colombia Colombia; 2 Departamento de Farmacia Universidad Nacional de Colombia Colombia Departamento de Farmacia, Universidad Nacional de Colombia Colombia; 3 Ministerio de Salud y Protección Social de Colombia Colombia Colombia Ministerio de Salud y Protección Social de Colombia Colombia

Existe consenso global sobre las dos maneras de obtener la autorización de comercialización de medicamentos: mediante un expediente completo para productos pioneros y mediante un expediente abreviado para competidores. Los principios científicos de la vía abreviada para la aprobación de biosimilares están descritos en el manuscrito (pág. 42, párrafo tercero).

También existe consenso en reconocer que la Administración de Medicamentos y Alimentos (FDA, por sus siglas en inglés) y la Agencia Europea de Medicamentos (EMA, por sus siglas en inglés) aceptan, en circunstancias específicas, que los ensayos clínicos confirmatorios pueden no ser necesarios cuando la eficacia y la seguridad se deduzcan claramente de la similaridad entre las características fisicoquímicas, la actividad o potencia biológica y los perfiles farmacocinéticos (PC) o farmacodinámicos (PD) del producto biosimilar y el de referencia. Zuluaga y Rodríguez sostienen, de manera equivocada, que la ruta abreviada de la comparabilidad colombiana (RACC) exime al fabricante de realizar estudios PC/PD. La guía de inmunogenicidad colombiana establece: *“En el caso de las proteínas terapéuticas competidoras se requiere que la similaridad de la eficacia y seguridad sean claramente deducidas desde las características fisicoquímicas, actividad biológica/potencia y los perfiles PC y/o PD del competidor y el producto de referencia…”*.

Colombia usa los mismos criterios que la FDA y la EMA para eximir los experimentos confirmatorios con seres humanos, pero propone una innovación regulatoria pues presenta estas exenciones como una tercera vía explícita (la RACC).

Las circunstancias específicas en las cuales un estudio clínico confirmatorio no será necesario van a ser más comunes que excepcionales, dada la velocidad de los avances analíticos y científicos en la caracterización de los bioterapéuticos, de modo que la ruta abreviada clásica para biosimilares se podría transformar en la RACC.

Comentarios específicos

El artículo presenta la justificación de la RACC y aclara que esta no elimina la obligatoriedad de estudios preclínicos o clínicos. En la RACC son explícitos los criterios y las circunstancias en las que, caso a caso, son posibles exenciones de experimentos con animales y seres humanos. Estos criterios siguen las tendencias internacionales.

El ejercicio de comparabilidad es escalonado y cada etapa se inicia en función de la incertidumbre residual y la relevancia clínica de las diferencias detectadas en la etapa previa. Se reconoce, por lo tanto, que podrían no existir dudas remanentes y. de esta manera, obviar el estudio clínico confirmatorio (estudio de equivalencia o de no inferioridad). La RACC dejó independiente esta posibilidad como alternativa regulatoria.

Están en lo correcto los profesores Zuluaga y Rodríguez en que Colombia es el único país en describir de forma independiente estos criterios y exenciones a través de una ruta específica. En otras regulaciones, estos criterios son parte de una única ruta de comparabilidad. Las razones para esta excepcionalidad se describen en el artículo, en especial en el último párrafo.

También hay coincidencia en que reducir no es lo mismo eximir. El interés del artículo es indicar cuándo y en qué circunstancias las regulaciones establecen la opción de reducir barreras técnicas y evitar la redundancia de experimentos con animales o seres humanos. La mayoría de los países analizados hablan de la reducción de los ensayos con seres humanos y animales y pocas mencionan la palabra “exención”; los países que permiten exenciones son Ecuador y Colombia. En Brasil, aunque nunca se usa la palabra “eximir”, incorpora la frase “cuando sean necesarios, los estudios fase I y fase II, no serán comparativos” (cuadro 3).

En Estados Unidos se usa la palabra exención (*waive*), aunque es claro que el requisito mínimo clínico es un estudio farmacociné-tico/farmacodinámico (pág. 45, párrafo 3).

Sobre la afirmación “los avances científicos y el conocimiento cada vez mayor sobre las proteínas permiten superar el paradigma de la comparabilidad”, si bien concedemos que podría ser exagerada, también advertimos que, en general, el “paradigma de la comparabilidad” se entiende como la necesidad de realizar un ejercicio completo de comparabilidad, desde la comparación analítica hasta la clínica (al menos un ensayo clínico comparativo de no inferioridad). Los autores afirman que “El paradigma de la comparabilidad hace énfasis en la necesidad de hacer ensayos clínicos confirmatorios, en todos los casos”. Esto no es necesariamente cierto, ni en Colombia ni en otros países. Por ello, sostuvimos que el “paradigma” estaría superado. En todo caso, aclaramos que la fase analítica deberá ser siempre comparativa. Es importante aclarar que, aun si se estima que es necesario un ensayo clínico, este no necesariamente tiene que ser comparativo (como establece Brasil).

Por último, en relación con los biocompetidores ya aprobados y que no han presentado problemas sobre su seguridad o eficacia, ¿por qué exigir un ejercicio completo de comparabilidad si, al estar en el mercado, la población expuesta al medicamento es mayor a la que participaría en cualquier ensayo clínico? ¿Qué información adicional, y más valiosa que la de poscomercialización, podría aportarse en cumplimiento de tal exigencia? Ninguna, desde el punto de vista epidemiológico o de salud pública. Esta medida constituiría una barrera innecesaria a la competencia.

